# Unique Luminescence of Hexagonal Dominant Colloidal Copper Indium Sulphide Quantum Dots in Dispersed Solutions

**DOI:** 10.1038/s41598-019-56762-8

**Published:** 2019-12-27

**Authors:** Samuel Jaeho Shin, Ja-Jung Koo, Jin-Kyu Lee, Taek Dong Chung

**Affiliations:** 1 Department of Chemistry, College of Natural Science, Seoul National University, Seoul, 08826 Republic of Korea; 2 Advanced Institutes of Convergence Technology, Suwon-Si, Gyeonggi-do 16229 Republic of Korea; 3 Present Address: Technology Research Centre, LG Chem, Seoul, 07796 Republic of Korea

**Keywords:** Inorganic chemistry, Materials chemistry, Materials for optics, Nanoscale materials

## Abstract

Luminescent hexagonal dominant copper indium sulphide (*h*-dominant CIS) quantum dots (QDs) by precursor-injection of mixed metal-dialkyldithiocarbamate precursors. Owing to the different reactivity of the precursors, this method allowed the CIS QDs to grow while retaining the crystallinity of the hexagonal nucleus. The photoluminescence (PL) spectra exhibited dual emission (600–700 nm red emission and 700–800 nm NIR emission) resulting from the combined contributions of the hexagonal (wurtzite) *h*-CIS and tetragonal (chalcopyrite) *t*-CIS QDs, i.e. the NIR and red emissions were due to the *h*-CIS QDs and coexisting *t*-CIS QDs (weight ratio of *h*-CIS/*t*-CIS ~ 10), respectively. The PL intensities of the *h*-CIS as well as *t*-CIS QDs were enhanced by post-synthetic heat treatment; the *t*-CIS QDs were particularly sensitive to the heat treatment. By separating *h*-CIS and *t*-CIS successfully, it was demonstrated that this phenomenon was not affected by size and composition but by the donor-acceptor pair states and defect concentration originating from their crystal structure. The *h*-dominant CIS QDs in this work provide a new technique to control the optical property of Cu-In-S ternary NCs.

## Introduction

Over the past two decades, research on semiconductor nanocrystals (NCs) has witnessed remarkable advances relating to their unique size-dependent optical properties^1^. Both fundamental and applied studies have been conducted with the aim of developing light-emitting properties and harvesting suitable materials for fluorescence bio-labelling^2–4^, light-emitting diodes^5,6^, solar cells^7–11^, etc. Although high-quality NCs have been researched to achieve tuneable colour, superior emission, and less photobleaching, there have been rising concerns regarding the materials containing toxic elements such as cadmium^1,12–14^ or lead^15–17^. I-III-VI_2_ semiconductor NCs are attractive because they pose low toxicity and have tuneable optical properties in the visible (VIS) to near-infrared (NIR) window, high absorption coefficients, large Stokes shifts, and relatively high photoluminescence (PL) quantum yields^18–20^. In this regard, copper indium sulphide (Cu_x_In_y_S_0.5×+1.5y_, CIS) has been considered a suitable candidate. Synthesis of luminescent CIS NCs has been one of the active research themes in the past decade, particularly for improving the luminescent properties and facilitating mass production^19,21–26^. Consequently, they pose the active potential to be utilized in various application areas such as bio-imaging^27–29^, optoelectronic devices^26,30–33^, and photovoltaics^34–36^.

Several factors affect the luminescence property of nanocrystals, such as the bandgap, size, composition, shape, and surface states^18^. The effect of the crystal structure is commonly overlooked owing to their minor contribution in most of the NCs even though it is widely accepted that the crystal structure is critical to the optoelectronic property and device performance^37–39^. However, this factor can be significant in two ways. First, in principle, the band structure of the semiconductor with a crystal structure is different, which can possibly lead to difference in the bandgap energy (*E*_g_) of the bulk semiconductor. Second, the defect states can vary significantly depending on their crystal structure and composition, which affects the luminescent properties of the NCs. The CIS quantum dots (QDs, 0D semiconductor nanocrystals, 3-dimensionally confined to be smaller than Bohr-exciton radius) are believed to support this consideration because the major contribution to the luminescence of CIS is not from its bandgap but from the donor-acceptor pair (DAP) generated from its inherent defects and the composition ratio of [Cu]/[In]. It is generally accepted that the major DAP states are formed by 2V_Cu_^−^ + In_Cu_^2+^ pairs, where In_Cu_^2+^ is the donor of the indium substituted at the Cu site and V_Cu_^−^ is the acceptor of the copper vacancy^26,40,41^. The other donor state of the sulphur vacancy (V_S_^2+^) can be systematically eliminated by using excess alkanethiols^26,40^. Furthermore, the defect states can be experimentally modulated by post-synthetic heat treatment, which is presumed to contribute to annealing^42^, atom mixing^43^, and defect curing^42^.

Thus far, conventional synthetic methods for realising high-quality luminescent CIS QDs employ indium (III) acetate, copper (I) iodide, and 1-dodecanethiol (1-DDT), and produce chalcopyrite (tetragonal) crystal structure of CIS (*t*-CIS), which is thermodynamically stable^25,26^. The study on luminescent CIS QDs with phases other than the tetragonal phase has been very limited compared to the active research on *t*-CIS. CIS can have other metastable structures such as wurtzite (hexagonal) and zinc blende (cubic), which are denoted as *h*-CIS and *c*-CIS, respectively. Recently, CIS QDs with metastable phases were successfully synthesised^38^. However, studies of their luminescent properties of *h*-CIS^44–47^ and *c*-CIS QDs^34,44,48^, are scarce with large size variation which is a significant disadvantage to discuss fundamental luminescent property in details. To the best of our knowledge, the luminescent property of *h*-CIS synthesized by topotactic partial Cu^+^ for In^3+^ exchange of hexagonal Cu_2-x_ is the report on size-controlled colloidal QDs^47^. However, this method cannot achieve In-rich CIS QDs although it serves as a guide for synthesising *h*-CIS from the hexagonal core.

In this work, we synthesised a luminescent *h*-dominant mixture of CIS QDs with diameter of a few nanometres by thermal decomposition of mixed metal-dialkyldithiocarbamate [M(R_2_DTC)_n_] precursors, in which the hexagonal core of Cu_2_S was formed prior to the incorporation of In_2_S_3_ clusters. The PL of the as-prepared CIS QDs was investigated as a function of the period of post-synthetic heat treatment. Interestingly, the PL spectra of the *h*-CIS and *t*-CIS QD mixtures showed two separate peaks, which responded to heat treatment with distinct sensitivities. We suspected that this was related to the difference in the defect states of CIS of different crystal structures; this conjecture was verified by case-by-case experiments.

## Results and Discussion

**Figure 1:**
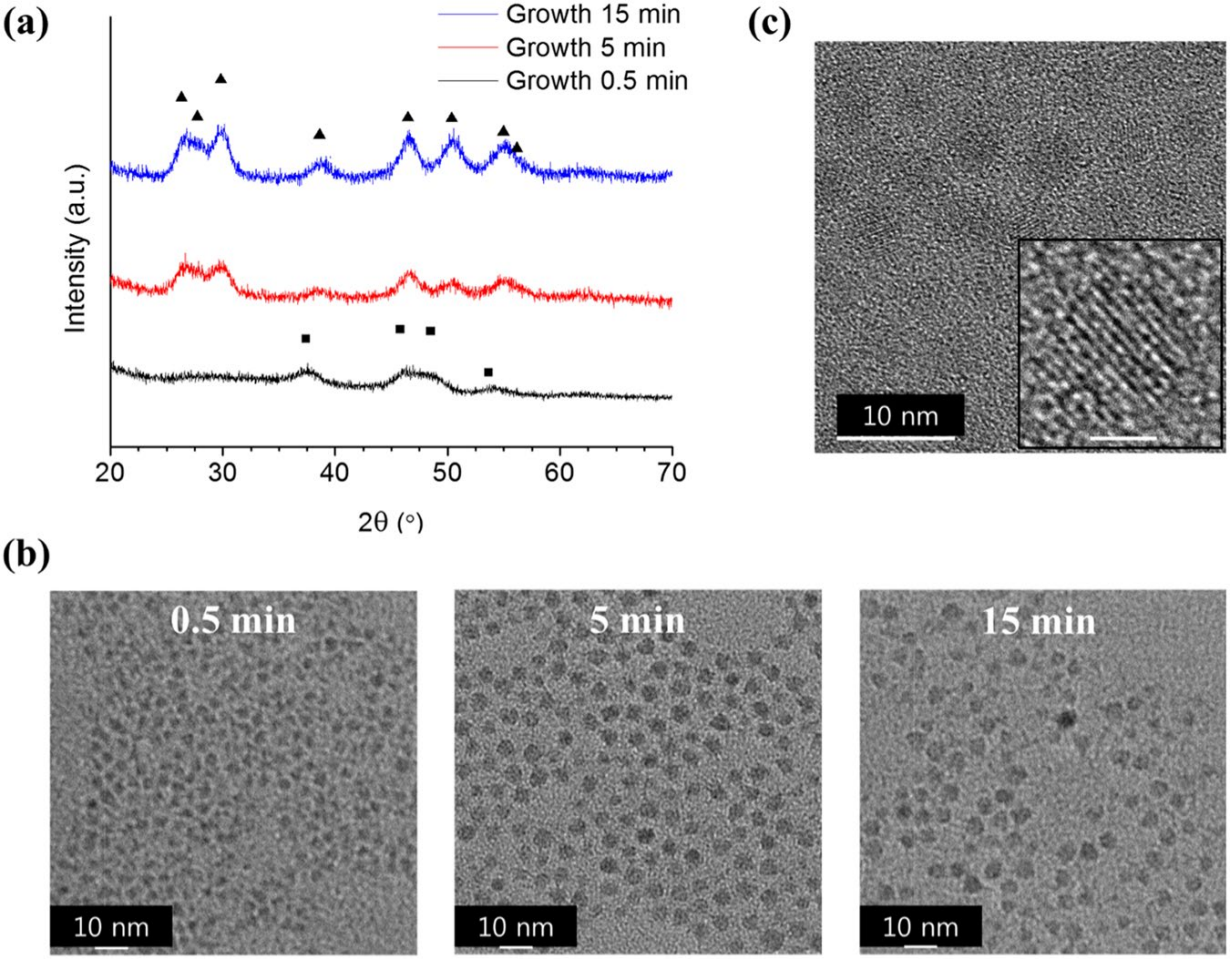
(**a**) XRD, (**b**) TEM images of *h*-dominant CIS QDs upon growth at 220 °C, and (**c**) HR-TEM image of the *h*-dominant CIS QDs after growth of 15 min; the rectangle (■) and triangle (▲) in (**a**) indicate the peaks from hexagonal chalcocite Cu_2_S (■ ICDD No. 00-026-1116) and *h*-CIS (▲ ICDD No. 01-077-9459), respectively. The scale bar of the inset of (**c**) is 2 nm.

In this work, we successfully synthesised *h*-dominant CIS QDs of approximately 4-nm diameter (Fig. 1) by a modified procedure of the method proposed by D. Pan *et al*.^38^. We employed the precursor-injection method for the synthesis, in which the size of the QDs was determined in the early nucleation stage. The size of the QDs slightly increased by less than 1 nm upon growth (Figs. 1b,c and S1). The crystal structures were characterised by XRD (Fig. 1a) and the [Cu]/[In] ratio obtained by inductively coupled plasma atomic emission spectroscopy (ICP-AES; Table 1). Both measurements confirmed the growth from hexagonal Cu_2_S (chalcocite, ICDD No. 00-026-1116) to hexagonal CIS (wurtzite, ICDD No. 01-077-9459). The impure crystal phases will be discussed in later paragraphs. Presumably, owing to the different reactivity of the precursors (Table S2), (Cu_2_S)_n_ clusters were dominantly generated when compared with the (In_2_S_3_)_n_ clusters, as they are nucleated faster to form hexagonal Cu_2_S crystal seeds in the early nucleation stage. Because the lattice mismatch between Cu_2_S and In_2_S_3_ is very small^43^, the Cu_2_S and In_2_S_3_ clusters were incorporated in the seeds without disturbing the crystal structure of the nucleus, resulting in *h*-CIS. In the superionic conducting state of Cu_2_S, ions are mobile in the nanostructure at the temperature prevailing during synthesis, indicating that fast alloying of components has occurred^43^ instead of the core/shell structure. The detailed structure characterisation is described in the later paragraphs. The slight excess of S content was ascribed to 1-DDT capping ligands, as reported by many other groups^23,25,26^.

**Table 1:** [Cu]/[In] ratios and molecular formulas of *h*-dominant CIS QDs after growth, based on ICP-AES results.

	0.5 m	1 m	3 m	5 m	15 m
Molecular Formula	Cu_3.99_In_0.24_S_2_	Cu_2.98_In_0.32_S_2_	Cu_1.41_In_0.97_S_2_	Cu_1.24_In_1.01_S_2_	Cu_0.97_In_0.83_S_2_
[Cu]/[In]	16.663	9.305	1.462	1.229	1.171

This dataset does not contain the average values. A single dataset was used owing to the inaccuracy of the numerical value but the data show a consistent trend of the compositional change in the early stage (0.5–5 min).

**Figure 2:**
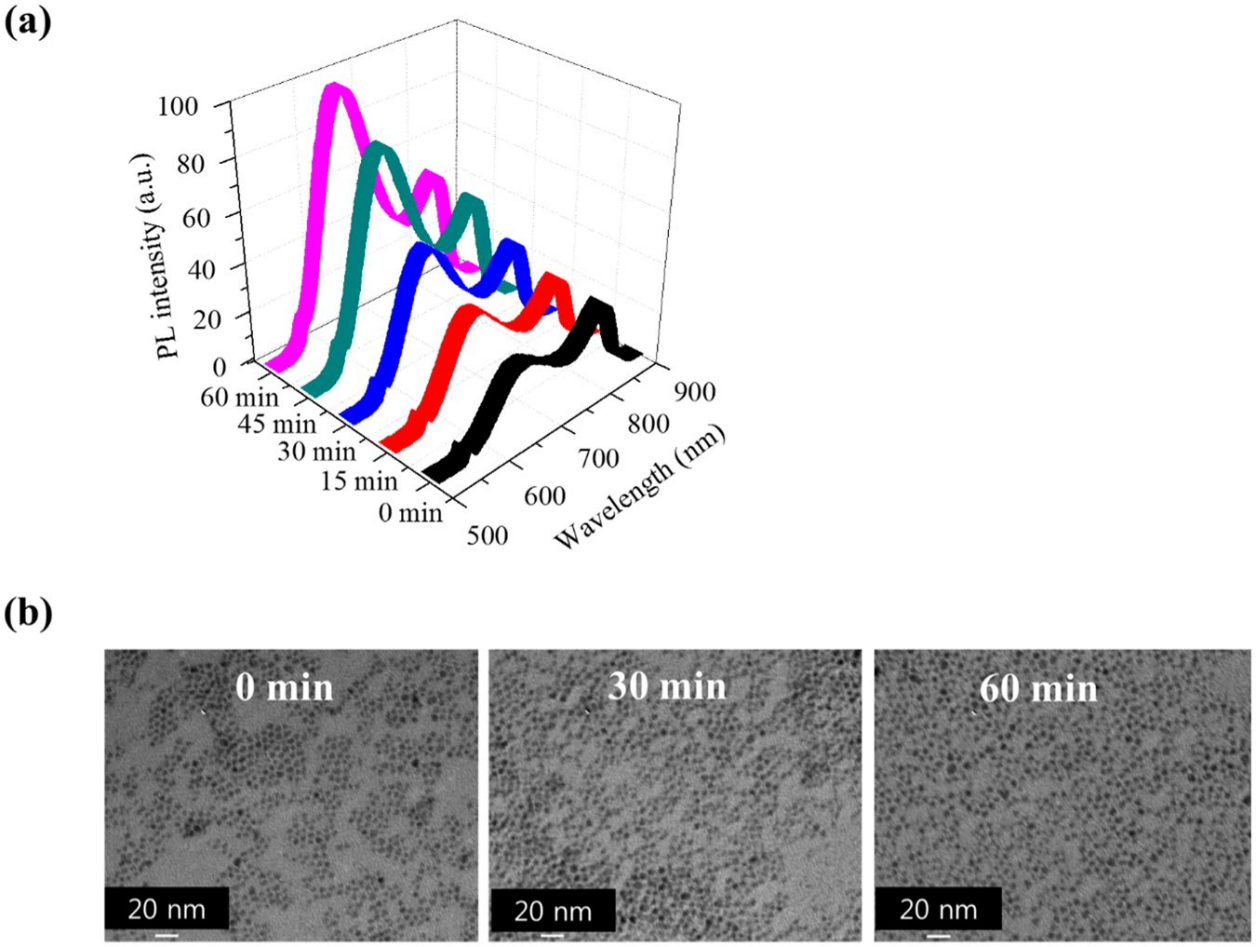
(**a**) PL spectra and (**b**) TEM images of *h*-dominant CIS QDs. The red-emission (600–700 nm) remarkably increased upon post-synthetic heat treatment whereas the NIR emission around 800 nm increased slightly. No significant change in size was observed upon heat treatment (see details in Fig. S3).

However, the PL spectra of the prepared CIS QDs showed a doublet, i.e. asymmetric red emission (600–700 nm) and NIR emission (700–800 nm) (0 min in Fig. 2 and Fig. S2). This does not follow the prediction by the ordinary QD growth mechanism based on the La Mer model, i.e. a broad emission. The overall PL intensities of CIS QDs increased upon post-synthetic heat treatment at 180 °C (Fig. 2). The spectra show the appearance of red emission in addition to NIR emission (Fig. 2a). The heat treatment did not significantly affect the size (Fig. 2b and Fig. S3) or the [Cu]/[In] ratio (Table 2), showing that no significant Ostwald ripening or compositional change occurred. This may be attributed to the structural or atominc displacement among the nanostructure of each CIS QDs. However, no such change was observed in the results of both XRD and medium-energy ion scattering (MEIS), details of which are described in the Supplementary Information (Fig. S4). This result is similar to but more pronounced than that observed in post-synthetic heat treatment of AgInS_2_ and ZnS-AgInS_2_ QDs by T. Torimoto *et al*.^42^. They observed a large increase in the PL intensity and slight blue shift of the PL peak, and reported that this phenomenon could be attributed to the change in the defect concentration of I-III-VI_2_ QDs, which is not sufficient to explain the results observed in this work. We believe that the crystal structure may have caused these observations and is discussed in the later paragraphs.

**Table 2:** [Cu]/[In] ratio of *h*-dominant CIS QDs upon heat treatment shown in Fig. 2.

	0 m	15 m	30 m	45 m	60 m
[Cu]/[In]	1.262	1.233	1.225	1.180	1.179
			Average	1.226 $\pm$ 0.041	

**Figure 3:**
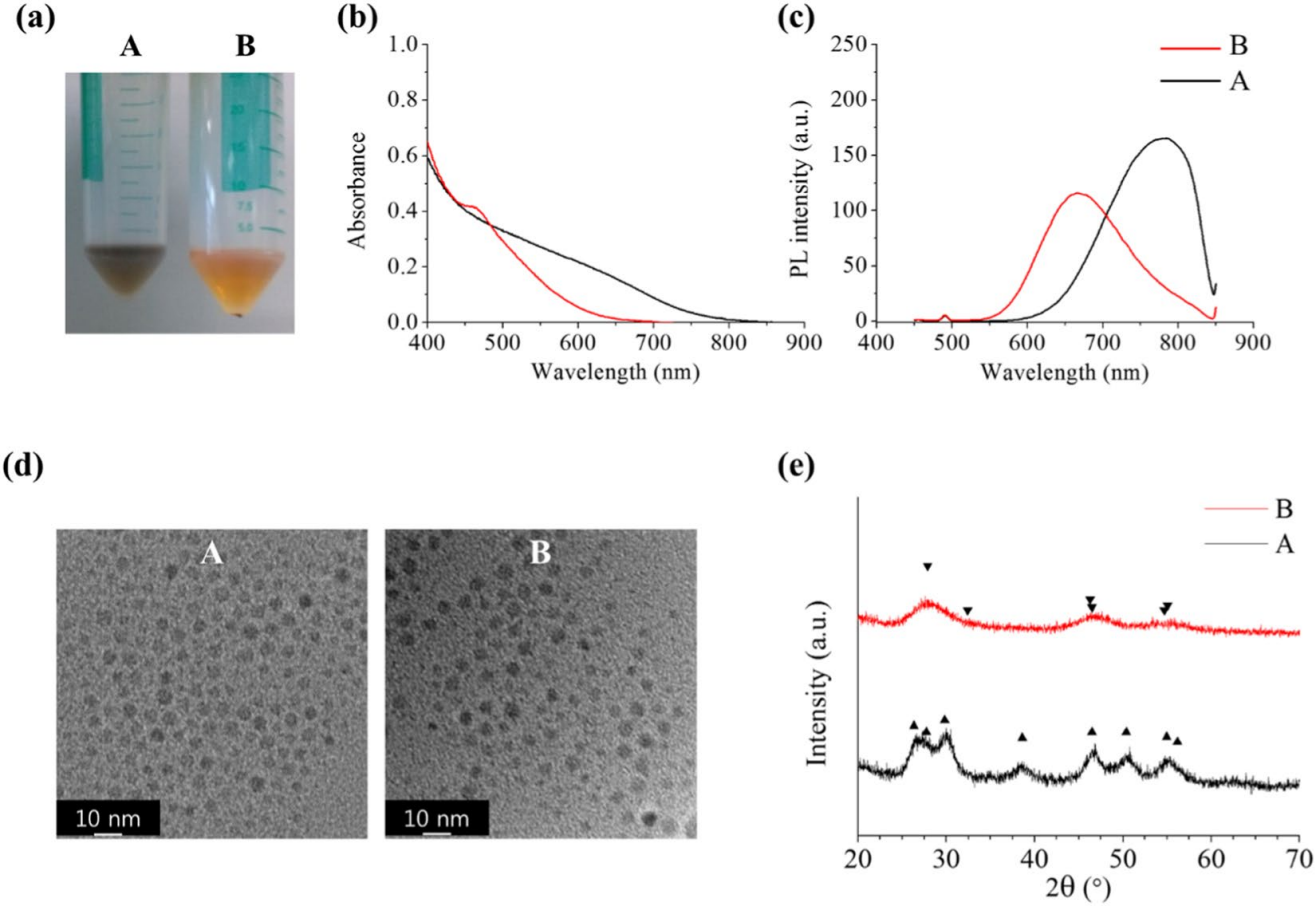
(**a**) Two samples (A and B) separated from *h*-dominant CIS QDs dispersed in hexane which was centrifuged in different manners. (**b**) UV-VIS absorption spectra, (**c**) PL spectra, (**d**) TEM images, and (**e**) XRD of separated CIS QDs; the triangle (▼) and inverted triangle (▼) indicate the peaks of *h*-CIS (▼ ICDD No. 01-077-9459) and *t*-CIS (▼ ICDD No. 01-081-9515), respectively.

We investigated further into the reasons that caused the above observations. We separated the *h*-dominant CIS QDs by centrifugation into samples A and B. The experimental details are described in the Supplementary Information. Based on several measurements, it was determined that the distinctive differences between them were the composition and crystal structure, which reflect their optical properties (Fig. 3 and Table 3). The sample A was Cu-rich CIS and showed a clear hexagonal structure (ICDD No. 01-077-9459) whereas the sample B was In-rich CIS characterised as a tetragonal structure (ICDD No. 01-081-9515). The PL spectra of the samples A and B were in the NIR and red range, respectively, with the weight ratio of A/B ~ 10. Their sizes were similar (Fig. 3d and S5) but the density differed with the composition (Cu_2_S: 5.6 g/cm^3^ and In_2_S_3_: 4.9 g/cm^3^). The bandgap of CIS reportedly increases with increase in the In content^26^, implying the possibility that the dual emission of the CIS QDs was attributed to compositional variation which is the [Cu]/[In] ratio. However, the characteristic doublet in PL spectra appears too conspicuous to attribute the dual emission to the change in the [Cu]/[In] ratio by considering Vegard’s law, which states that the bandgap is linearly related to the concentration of the constituent elements, assuming a consistent single crystal structure. Therefore, it should be rational to seek a different factor that could have caused this phenomenon.

**Table 3:** [Cu]/[In] ratio and the molecular formula of separated CIS QDs.

	[Cu]/[In]	Molecular Formula
Fully precipitate	1.197	Cu_1.32_In_1.10_S_2_
A	1.189	Cu_1.37_In_1.16_S_2_
B	0.830	Cu_0.83_In_1.01_S_2_

**Figure 4:**
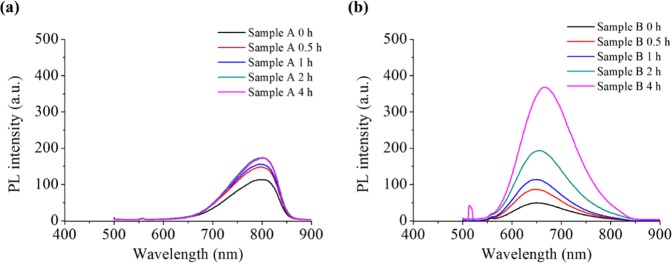
Effect of post-synthetic heat treatment on PL spectra of (**a**) A (*h*-CIS) and (**b**) B (*t*-CIS) QDs isolated from *h*-dominant CIS QDs.

As discussed earlier, the luminescent property of the CIS QDs originates from both the bandgap and the DAP states. The major contribution to the luminescence of CIS is generally accepted to be from the DAP states of the 2V_Cu_^−^ + In_Cu_^2+^ pairs. These states may vary with the crystal structure. In addition, the DAP states are reported to be modulated by post-synthetic heat treatment^42,43^. Therefore, we conducted heat treatment for samples A and B and found that the PL of both samples was enhanced by the heat treatment. However, the degree of enhancement was remarkably different for the two crystal structures (Fig. 4). The PL enhancement of sample B (*t*-CIS) was much larger than that of sample A (*h*-CIS), which can be attributed to the significant difference in the change in defect concentration. Such enormous PL enhancement of the tetragonal phase I-III-VI_2_ QDs is supported by a past study^42^. This shows that the dual emissions, including asymmetric Gaussian PL with red-tailing, can result from variation in the defect concentration, which can be hardly controlled during synthesis.

As mentioned earlier, the minor contribution of the PL from the bandgap of the CIS QDs must be considered. The *E*_g_ of the bulk semiconductor of *t*-CIS (1.45–1.53 eV, 810–855 nm)^18^ is generally reported to be larger than that of *h*-CIS (1.40–1.47 eV, 844–886 nm)^38,49,50^. It is difficult to estimate the increment in *E*_g_ with decrease in size due to quantum confinement effect. However, this provides insight into the wavelength difference between the two observed doublets (approximately 120–140 nm), which widened with quantum confinement in comparison with the bulk semiconductor (approximately 10–70 nm).

The reason for the synthesis of the mixed crystal structure in *h*-dominant CIS QDs is still not clearly understood. We believe this must be related to the crystal structure transformation based on the composition and atom (phase) mixing. It should be noted that the [Cu]/[In] ratio is dependent on the crystal structure. The XRD results from the CIS samples synthesised by changing the metal component ratio (Fig. S6) indicate that the crystal structure can be transformed to form either hexagonal or tetragonal structures depending on the ratio of the metallic components. The hexagonal phase is preferentially obtained with high [Cu]/[In] ratio whereas similar-to-tetragonal phase is likely to be formed with low [Cu]/[In] ratio. A similar phenomenon was observed in a previous study^26^. In the early nucleation and growth stage, the hexagonal structure of the (Cu_2_S)_n_ seed determines the crystal structure, as described earlier. It must be mentioned that from a thermodynamic perspective, the hexagonal structure is preferred in Cu_2_S whereas the tetragonal structure is preferred in In_2_S_3_. In addition, Cu_2_S phase is known to have low superionic transition temperature theoretically above 376 K (~100 °C)^43^. As In_2_S_3_ grows on CIS clusters, Cu_2_S can move freely among the nanostructure, resulting in alloying (atom mixing) as well as possible transformation to the crystal structure by distorting the anion framework during crystal growth and post-synthetic heat treatment. Such transformation from the metastable phase to the stable crystal structure was already observed for the similar MIn_2_S_4_ (M = Mn, Fe, Co) system by TG-DTA, XRD analysis, and calculation with the Vienna ab initio simulation package^51^.

The effect of the composition and growth of the shell on the optical property of CIS QDs is also critical. Therefore, In-rich (0.1: 0.3) *h*-CIS QDs (notation described in the Methods) were synthesised followed by post-synthetic heat treatment. It also showed a doublet of the PL as synthesized and the degree of PL enhancement was again stronger for red emission (600–700 nm) than NIR emission (700–800 nm) upon post-synthetic heat treatment without compositional change (Fig. S7a and Table S3). This can be explained by the difference in the crystal structure, defect states, and defect concentrations as discussed earlier. In addition, hexagonal (wurtzite) ZnS shell was introduced as the thermodynamically stable crystal structure to apply strain on the inner core of CIS and reduce the surface defect states by passivation. No dual emission was observed and the PL intensity increased with blue-shift upon post-synthetic heat treatment without composition change (Fig. S7b and Table S4). We believe that the crystal structure of CIS cannot be distorted by the post-synthetic heat treatment due to the strain acting by the ZnS shell, thus retaining the crystal structure. Therefore, the change in the defect concentration only influenced the optical property of *h*-CIS/ZnS, which agrees with the results of the previous work on AgInS_2_ and ZnS-AgInS_2_ QDs by T. Torimoto *et al*.^42^.

In summary, luminescent *h*-dominant CIS QDs (0D) of 3–4-nm diameter were synthesised by utilising the different reactivity of the metal-dialkyldithiocarbamate precursors. The composition and crystal structure of the CIS QDs were analysed after growth. The compositional variation during synthesis affected the resulting crystal structures. Dual emission was observed in the PL spectra, which could be attributed to the combined contributions of the *h*- and *t*-CIS QDs. The mixture was separable; therefore, the optical properties of the *h*-CIS and *t*-CIS in the mixture were investigated after post-synthetic heat treatment. The PL of both *h*-CIS QDs and *t*-CIS QDs was enhanced by the heat treatment; the degree of enhancement was remarkably dependent on the crystal structures of the CIS QDs. The effect of heat treatment can be explained by the DAP states originating from the defects owing to difference in the crystal structures. The findings in this work provide valuable perspectives into the synthetic mechanism of *h*-CIS QDs as well as improve the understanding of the optical property of the Cu-In-S ternary system of a few nanoscales in terms of the unusual but important aspects of the crystal structure. Furthermore, this valuable information may be considered for many applications of CIS NCs such as optoelectronic devices, bio-imaging, and solar cells.

## Methods

### Chemicals

Diphenyl ether (DPE, 99%), 1-dodecanethiol (1-DDT, 98%), oleic acid (OA, 90%), octylamine (OcAm, 99%), and 1-octadecene (1-ODE, 90%) were purchased from Sigma-Aldrich; zinc dimethyldithiocarbamate (Zn(Me_2_DTC)_2_, 95%) was from Tokyo Chemical Industry, TCI. All chemicals were used as received without further purification.

### Preparation and characterization of precursors

Experimental details and characterization described in Supplementary Information (Tables S1 and S2).

### Synthesis of *h*-dominant CIS QDs by precursor-injection and their post-synthetic heat treatment

The procedure of synthesis was modified from the previous report^38^. All of these reactions were performed by standard Schlenk line method. Cu(Et_2_DTC)_2_ (copper diethyldithiocarbamate, x mmol), In((i-Pr)_2_DTC)_3_ (indium diisopropyldithiocarbamate, y mmol), 1-DDT (1 mL), and OA (0.25 mL) were mixed with 2 mL of DPE solvent (x + y = 0.4) in two-neck round-bottomed flask connected with a condenser and a thermocouple adapter. The components were not homogeneously mixed at room temperature. It was degassed at 60 °C under vacuum for 20 min. Switched to N_2_ atmosphere, it was heated to 120 °C for 10–20 min in order to obtain a clear homogeneous solution. Using a glass-syringe, the precursor solution was transferred to the 8 mL of preheated DPE solvent at 250 °C under N_2_ atmosphere by swift injection. The temperature of the hot solvent was dropped to approximately 220 °C and was kept for 15 min for growth. Growth step was terminated by taking away the flask from the heating mantle. It is denoted as (x: y) *h*-CIS QDs by their initial composition of the starting materials. Unless otherwise stated, *h*-dominant CIS QDs indicate the produced QDs starting from (0.2:0.2) as the initial composition.

The post-synthetic heat treatment was performed either by cooling without purification or heating to similar temperature after purification followed by re-dispersion in 1-ODE together with OA and 1-DDT surfactants. A small amount of aliquots were taken at a specific time in order to analyse the CIS QDs. Excess EtOH or acetone was added to the sampled aliquots, which were subsequently centrifuged for purification. The resulting QDs can be dispersed in nonpolar solvents such as hexane, toluene, and 1-ODE. A single purification step was enough for optical characterization, while at least 3 times were needed for TEM, XRD, and ICP-AES analyses.

### Optical characterization

The absorption and photoluminescence (PL) spectra of the purified QDs dispersed in hexane were acquired by Scinco S-3100 UV-VIS spectrometer and Jasco FP-6500 spectrofluorometer, respectively. The optical density of the samples was adjusted referring to the absorbance at 480 nm wavelength. The excitation wavelength was fixed at 480 nm for PL measurement.

### Transmission electron microscopy (TEM)

For TEM analysis, the QDs dispersed in toluene were drop-casted onto carbon-coated copper grids. Either JEOL JEM-2100 or Hitachi 7600 was used, where the accelerating voltages were 200 and 100 kV for former and latter, respectively. High-resolution TEM (HR-TEM) analysis was performed by JEOL JEM-2100F equipped with field emission gun working at 200 kV. For size determination, 100 to 150 particles in TEM images were measured and statistically treated (see details in Supplementary Information).

### ICP-AES analysis

Few milligrams of purified QDs were completely digested in 1 mL of *aqua regia* then diluted with distilled water to 20 mL. The measurement was performed by Perkin-Elmer Optima 4300 DV. The composition of QDs was mostly determined by this technique, otherwise stated.

### Powder X-ray diffraction (XRD)

Powder XRD was acquired with a Bruker New D8 Advance diffractometer in reflection geometry using Cu Kα1 radiation. 2θ range of 10–90° was scanned at 5°/min.

## Supplementary information

Supplementary Information.

## Data Availability

All data needed to evaluate the conclusions in the paper are present in the manuscript and/or the Supplementary Information. Additional data related to this paper may be requested from the authors.
