# Supplementary material for: Unique Luminescence of Hexagonal Dominant Colloidal Copper Indium Sulphide Quantum Dots in Dispersed Solutions
Source: Sci Rep. 2019 Dec 27;9:20144. doi: 10.1038/s41598-019-56762-8 (PMC6934773; doi:10.1038/s41598-019-56762-8)
Supplement: Supplementary file 1 — Supplementary Information. [file 41598_2019_56762_MOESM1_ESM.docx]

Supplementary Information

**Unique Luminescence of Hexagonal Dominant Colloidal Copper Indium Sulphide Quantum Dots in Dispersed Solutions**

Samuel Jaeho Shin^1^, Ja-Jung Koo^1^, Jin-Kyu Lee^1†^, and Taek Dong Chung^1,2★^

^1^ Department of Chemistry, College of Natural Science, Seoul National University, Seoul, 08826, Republic of Korea.

^2^ Advanced Institutes of Convergence Technology, Suwon-Si, Gyeonggi-do 16229, Republic of Korea.

^†^ Currently moved to Future Technology Research Centre, LG Chem, Seoul, 07796, Republic of Korea.

^★^ To whom correspondence should be addressed. E-mail: tdchung@snu.ac.kr

**Methods**

**Chemicals**

Indium(III) chloride (InCl_3_, 98 %), copper(II) chloride dihydrate (CuCl_2_∙2H_2_O, 99 %), sodium diethyldithiocarbamate trihydrate (NaS_2_CNEt_2_, ACS reagent), and diisopropylamine ((*i*-Pr)_2_NH, 99 %), were purchased from Sigma-Aldrich; carbon disulphide (CS_2_, 98 %) was from Kanto Chemicals; zinc dimethyldithiocarbamate (Zn(Me_2_DTC)_2_, 95 %) was from Tokyo Chemical Industry, TCI. All chemicals were used as received without further purification.

**Synthesis of Metal N,N-dialkyldithiocarbamate Precursors (M(R_2_DTC)_n_, M = In(III) or Cu(II), R = Et or *i*-Pr) and Characterisation of Their Structure and Thermal Behavior**

NaS_2_CNEt_2_ was used as purchased. Sodium diisopropyldithiocarbamate, NaS_2_CN(*i*-Pr)_2_, was synthesised in a similar manner of the reported case earlier^1^. 10 mmol (*i*-Pr)_2_NH was dissolved in 10 mL ice-cold acetone. The ice-cold solution containing 30 mmol CS_2_ in 10 mL acetone was added drop-wise to the amine solution. After stirring in ice-bath for 2 h, 10 mmol of saturated NaOH aqueous solution (~ 0.53 mL) was added and stirred 4 h additionally at room temperature. Evaporation gave the crude product as a pale yellow powder, which was directly used to prepare the precursors without further purification.

The M(R_2_DTC)_n_ were prepared using a similar method reported in the literature^2^. Equimolar amounts of aqueous metal chlorides, CuCl_2_∙2H_2_O or InCl_3_, were mixed with corresponding ligands in distilled water. The brown (copper) and white (indium) precipitate immediately occurred and further stirred for 1 h. The precipitates were filtered, washed with an excess amount of distilled water, and dried in 80 – 100 ˚C vacuum oven for 2 – 3 h. Cu(Et_2_DTC)_2_, Cu((*i*-Pr)_2_DTC)_2_, In(Et_2_DTC)_3_, and In((*i*-Pr)_2_DTC)_3_ gave good yields, above 93%. The molecular formula of the prepared precursors was confirmed by elemental analysis using Thermo Electron Corp. Flash EA 1112 elemental analyser (the metal content was determined by subtracting the weights of C, H, N, and S from total)^3^. Their thermal behavior was measured by both DSC-TGA and pyrolysis in solution. DSC-TGA measurement was performed by loading few milligrams of precursors into ceramic pan, equilibrated at 100 ˚C for 30 min then ramped to 700 ˚C with the rate of 10 ˚C /min at N_2_ atmosphere using TA Instruments SDT Q600. Pyrolysis in solution was observed starting with the preparation of mixtures described in *h*-CIS synthesis with single precursors only. At N_2_ atmosphere by standard Schlenk line method, the solution was heated starting from 150 ˚C. The temperature was kept for 1 h, then raised by 10 ˚C, step by step. In case of In((*i*-Pr)_2_DTC)_3_, which the solution was colourless transparent, turned to slight yellow when started to decompose. Cu(Et_2_DTC)_2_ mixture, on the other hand, was clear dark brown and Cu_2_S was also dark brown solution. Thus, it was impossible to observe abrupt colour change. Therefore, a few aliquots were taken at each step, adding an insoluble solvent to see if the precipitate occurred.

**Medium Energy Ion Scattering (MEIS)**

MEIS was taken to observe the composition among the nanocrystals since Collection of scattered intensity gives information about where the atoms are relatively positioned inside the particles^4,5^.

MEIS spectra were taken using TOF-MEIS K-120 with He^+^ ion acceleration energy of 100 keV and 135.75˚ scattering angle for high atomic resolution. The samples dispersed in hexane were diluted to the appropriate concentration in order to form monolayer assembly on the substrate. It was dropped and spread on diamond-like carbon.

**Separation of A and B from *h*-Dominant CIS QDs by Centrifugation**

2 equivalent volume of EtOH was added to the as-synthesised solution and centrifuged them at 4,000 rpm. Sample A was obtained by taking the sediment at the bottom. On the other hand, sample B was prepared by decanting the previous supernatant, adding a large excess amount of EtOH, and centrifuging again at 17,000 rpm. This process is similar to the so-called size-selection experiment of nanoparticles^6^, but the separation was based on density difference upon composition difference. The detailed explanation is described in the manuscript and Fig. S5.

**Results**

Table S1. Elemental analysis results for M(R_2_DTC)_n_ compounds, indicated in wt %.

|  | **Cu** | **In** | **N** | **C** | **H** | **S** |
| --- | --- | --- | --- | --- | --- | --- |
| Cu(Et_2_DTC)_2_ | 18.7477 | - | 7.8427 | 33.7802 | 5.4278 | 34.2016 |
| Cu((*i*-Pr)_2_DTC)_2_ | 14.2245 | - | 6.7223 | 40.1897 | 6.6714 | 32.1921 |
| In(Et_2_DTC)_3_ | - | 17.6721 | 7.6931 | 32.5476 | 5.4191 | 36.6681 |
| In((*i*-Pr)_2_DTC)_3_ | - | 16.3111 | 6.5954 | 39.1669 | 6.5284 | 31.3982 |

Table S2. Thermal behavior of M(R_2_DTC)_n_ compounds measured by both DSC-TGA and thermal decomposition in solution.

| **Precursor** | **Weight Loss (%)** | **Temperature Range**  **by TGA (**°**C)** | **Temperature Range by**  **Pyrolysis in Solution (**°**C)** |
| --- | --- | --- | --- |
| Cu(Et_2_DTC)_2_ | 78.28 | 234.22 – 403.44 | 160 – 180 |
| In((*i*-Pr)_2_DTC)_3_ | 72.12 | 261.16 – 349.41 | 210 – 230 |


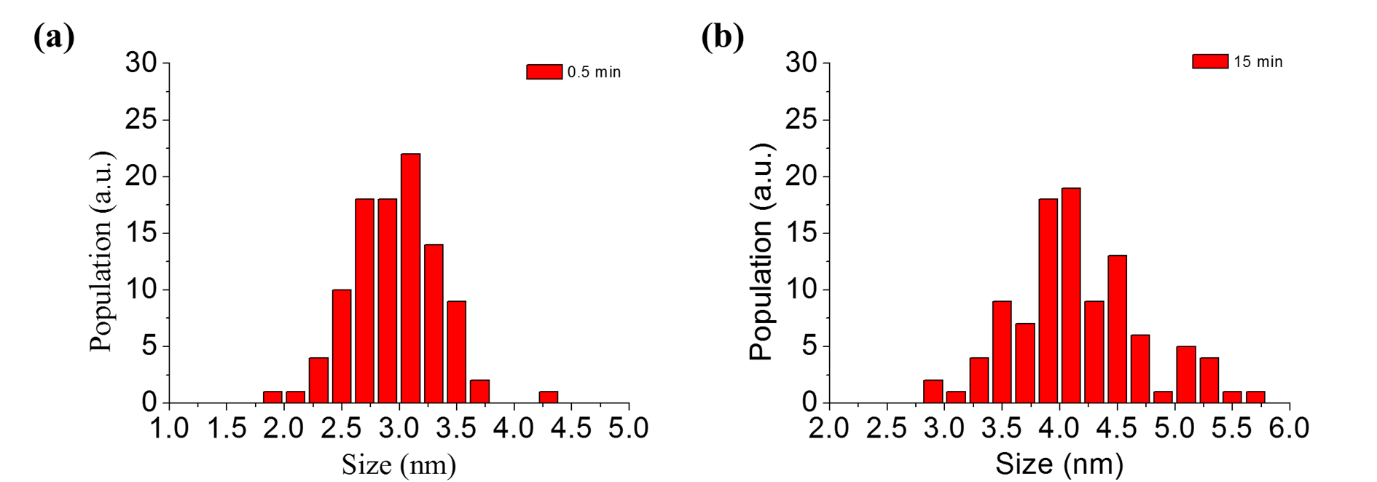


**Figure S1.** Histogram of *h*-dominant CIS QDs upon growth at (a) 0.5 min and (b) 15 min. The determined sizes were 2.86 ± 0.38 nm and 4.06 ± 0.57 nm respectively.


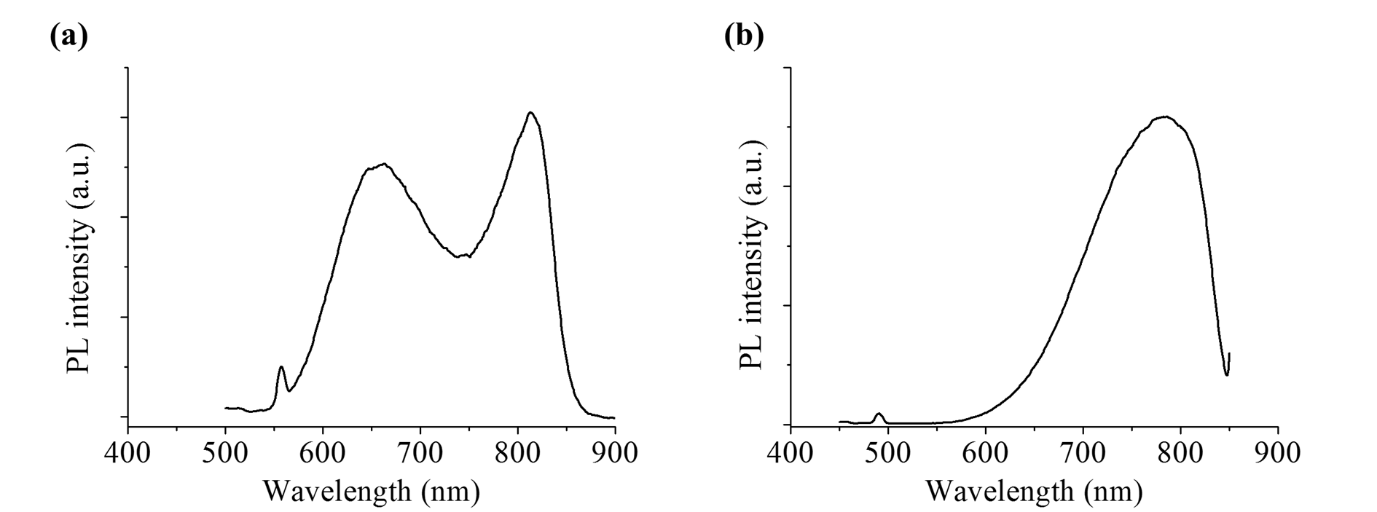


**Figure S2.** PL spectra of *h*-dominant CIS QDs either (a) dual emission or (b) asymmetric Gaussian with red-tailing. The doublet mostly occurred as synthesised while the asymmetric Gaussian with red-tailing was seldom.


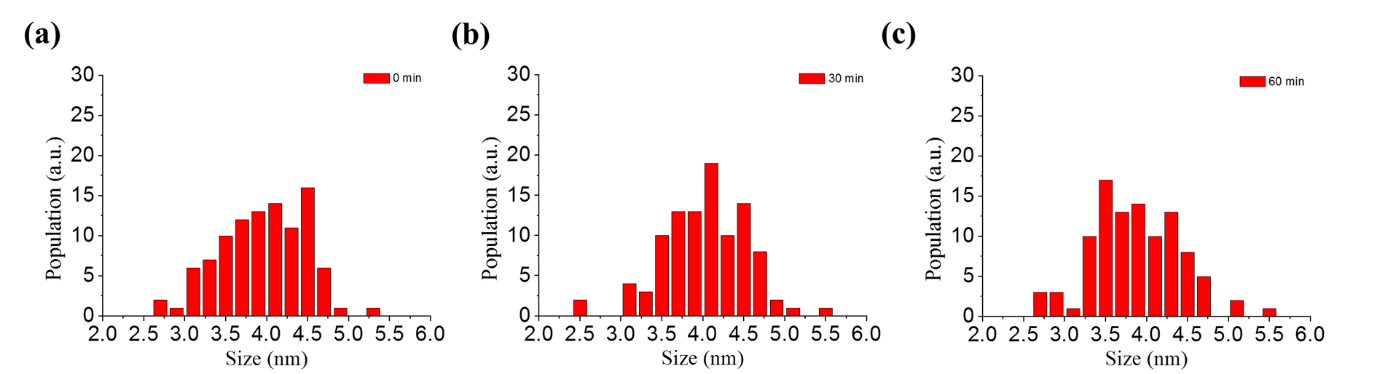


**Figure S3.** Histogram of *h*-dominant CIS upon post-synthetic heat-treatment at (a) 0 min, (b) 30 min, and (c) 60 min. The determined sizes were 3.76 ± 0.50 nm, 3.83 ± 0.52 nm, and 3.68 ± 0.54 nm respectively.


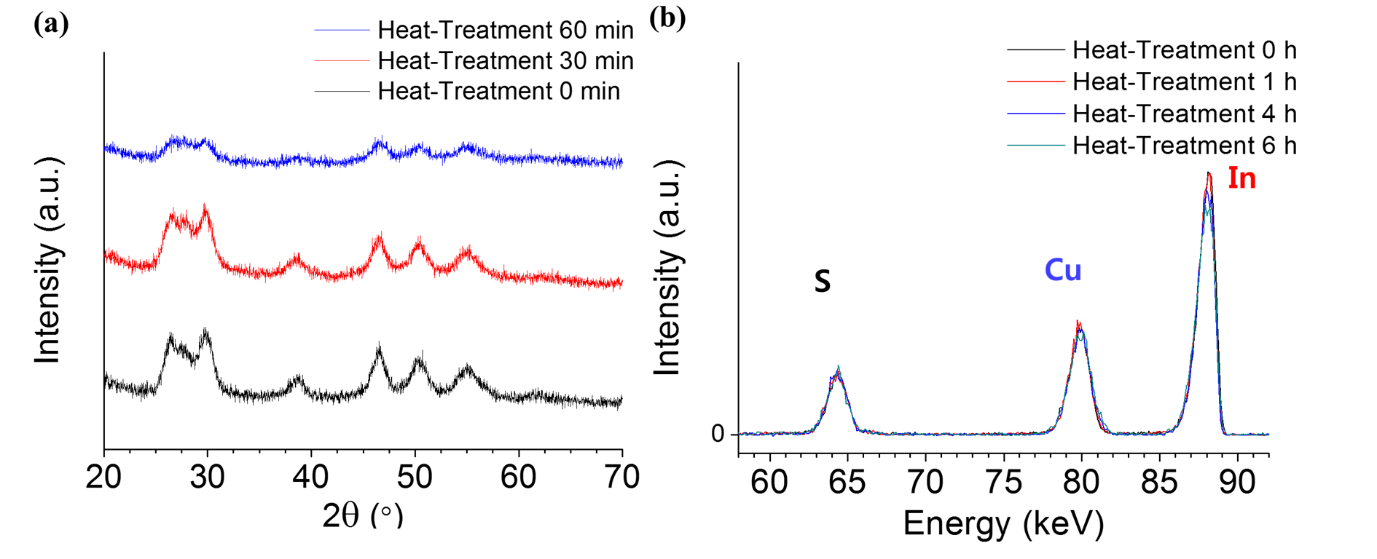


**Figure S4.** (a) XRD, (b) MEIS of *h*-dominant CIS upon post-synthetic heat treatment at 180 °C; the *h*-CIS (ICDD No. 01-077-9459) was retained upon such treatment.

MEIS spectra were obtained by collecting scattered He^+^ ion at a specific angle where the detector was installed. Collection of scattered intensity gives information about where the atoms are relatively positioned inside the particles^4,5^. The absence of significant change (Fig. S4b) tells no atomic displacement inside the NCs upon post-heat treatment. Being consistent with the XRD result (Fig. S4a), MEIS spectra can lead us to the conclusion that the synthesised CIS had homogeneous atomic distribution among nanostructure rather than core/shell.


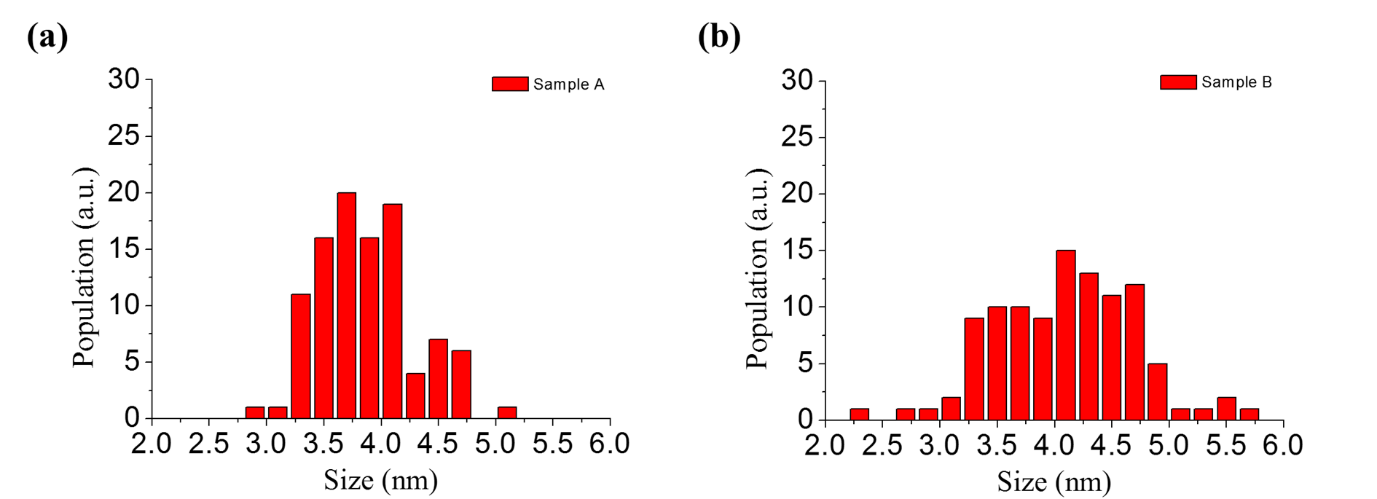


**Figure S5.** Histogram of the samples A and B separated from *h*-dominant CIS QDs: (a) sample A and (b) sample B. The determined sizes were 3.76 ± 0.40 nm, and 3.99 ± 0.61 nm respectively.

The weight ratio of A/B was approximately 10. The optical properties of sample A and B were presented in Figs. 3a-c. While this procedure is similar to so-called size-selection experiment of nanoparticles^6^, it turned out that there was significant distinction in [Cu]/[In] rather than in size (sample A: 3.76 ± 0.41 nm, sample B: 3.99 ± 0.61 nm. See details in Fig. S5) as shown in Fig. 3d and Table 3. In other words, preferentially centrifuged sample A was not due to size but density difference of QDs. This is reasonable considering the densities of Cu_2_S (5.6 g/cm^3^) and In_2_S_3_ (4.9 g/cm^3^).


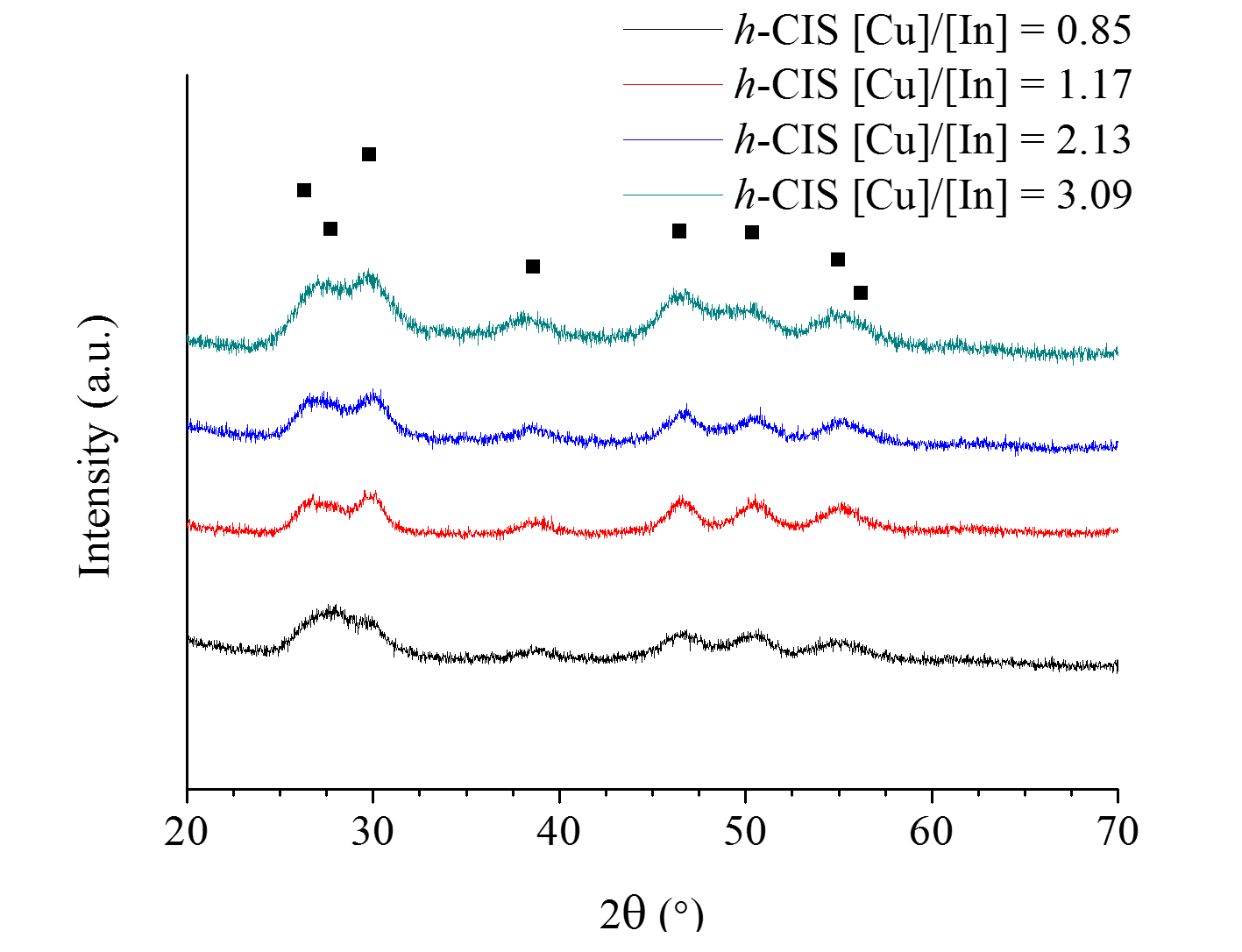


**Figure S6.** XRD of *h*-dominant CIS varying [Cu]/[In] ratio. The ratios from the starting material were (0.3 : 0.1), (0.25 : 0.15), (0.2 : 0.2), (0.1 : 0.3) from bottom to top of XRD spectra, respectively; the rectangle (■) indicates the peaks from *h*-CIS. (■ ICDD No. 01-077-9459) The shape and 2θ peak position slightly shift as [Cu]/[In] ratio varies. In case of [Cu]/[In] = 0.85, it was very similar to the XRD pattern of *t*-CIS (Fig. 3e, ICDD No. 01-081-9515), indicating the chance of distortion from *h*-CIS to *t*-CIS.


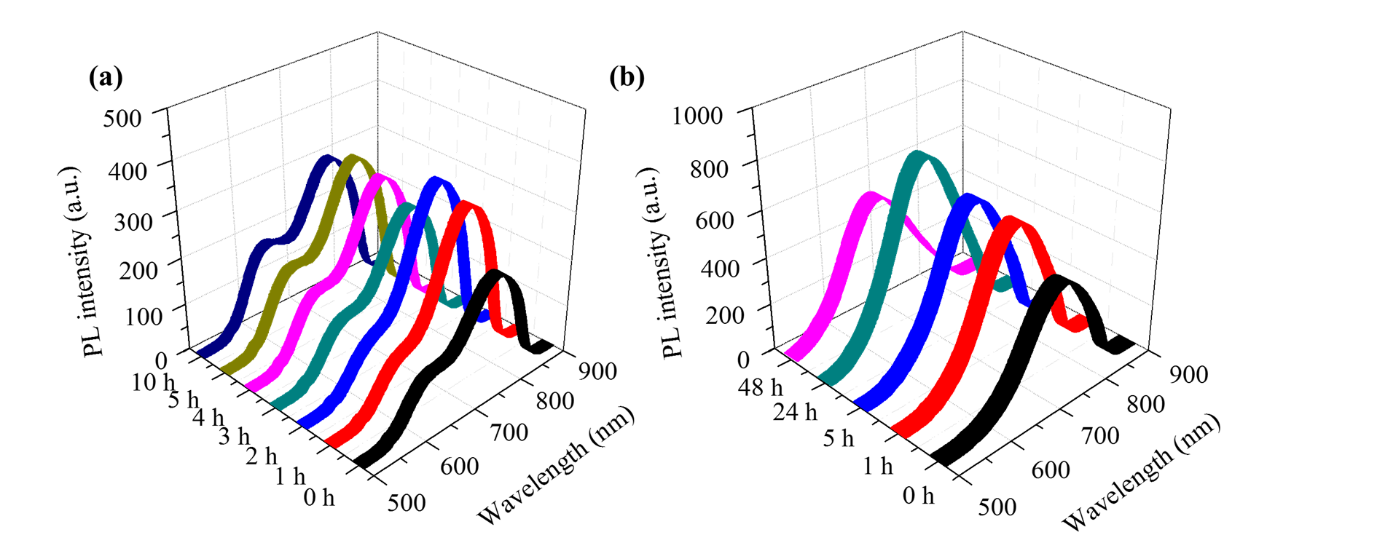


**Figure S7.** PL spectra of (a) (0.1 : 0.3) *h*-CIS and (b) (0.1 : 0.3) *h*-CIS/ZnS upon post-synthetic heat treatment after purification.

Table S3. [Cu]/[In] ratios and molecular formula of (0.1 : 0.3) *h*-CIS upon post-synthetic heat treatment after purification.

|  | **0 h** | **1 h** | **2 h** | **3 h** | **5 h** | **10 h** |
| --- | --- | --- | --- | --- | --- | --- |
| [Cu]/[In] | 0.823 | 0.859 | 0.913 | 0.905 | 0.793 | 0.816 |
|  |  |  |  | Average | 0.859 $\pm$ 0.050 | |

Table S4. [Cu]/[In] ratios and molecular formula of (0.1 : 0.3) *h*-CIS/ZnS upon post-synthetic heat treatment after purification.

|  | **0 h** | **1 h** | **5 h** | **24 h** | **48 h** |
| --- | --- | --- | --- | --- | --- |
| [Cu]/[In] | 1.013 | 1.018 | 1.050 | 1.080 | 1.128 |
| [Zn]/[Cu] | 0.445 | 0.465 | 0.380 | 0.396 | 0.409 |
| [M]/[S] | 0.944 | 0.865 | 0.866 | 0.951 | 0.995 |
|  |  | Average | [Cu]/[In] | 1.058 $\pm$ 0.048 | |
|  |  |  | [Zn]/[Cu] | 0.419 $\pm$ 0.035 | |
|  |  |  | [M]/[S] | 0.924 $\pm$ 0.057 | |

[M] = [Cu] + [In] + [Zn]


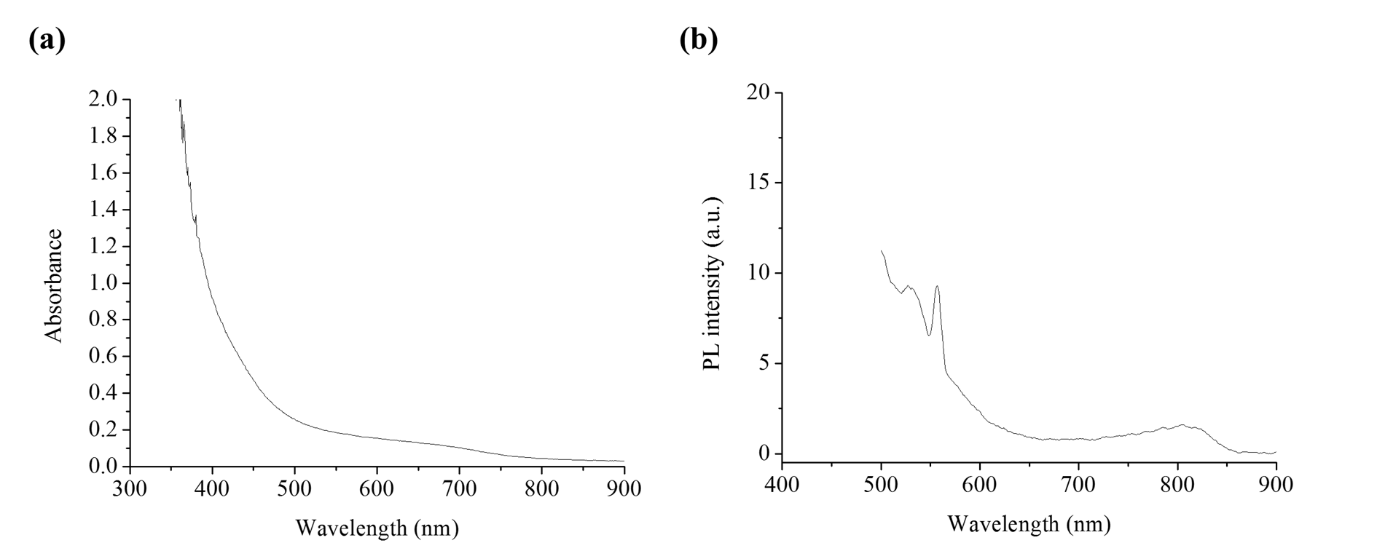


**Figure S8.** (left) UV-Visible and (right) PL spectra (*λ*_ex_ = 480 nm, absorption spectrum fixed to *A*_480nm_ = 0.35) of precursor solution in hexane.

**References**

1 Ono, D., Bragdon, J. & Jaeger, D. A. Synthesis and characterization of dithiocarbamate surfactants. *Colloid. Surf. A Physicochem. Eng. Asp.* **308**, 141-146 (2007).

2 Jung, Y. K., Kim, J. I. & Lee, J. K. Thermal decomposition mechanism of single-molecule precursors forming metal sulfide nanoparticles. *J. Am. Chem. Soc.* **132**, 178-184 (2010).

3 Oliveira, M. M. *et al.* N,N '-dialkyldithiocarbamate chelates of indium(III): alternative synthetic routes and thermodynamics characterization. *Thermochim. Acta* **328**, 223-230 (1999).

4 Sortica, M. A. *et al.* Structural characterization of CdSe/ZnS quantum dots using medium energy ion scattering. *Appl. Phys. Lett.* **101**, 023110 (2012).

5 Jung, K. W. *et al.* Quantitative compositional profiling of conjugated quantum dots with single atomic layer depth resolution via time-of-flight medium-energy ion scattering spectroscopy. *Anal. Chem.* **86**, 1091-1097 (2014).

6 Murray, C. B., Norris, D. J. & Bawendi, M. G. Synthesis and characterization of nearly monodisperse CdE (E = S, Se, Te) semiconductor nanocrystallites. *J. Am. Chem. Soc.* **115**, 8706-8715 (1993).
